# The National Association of Dental Faculties

**Published:** 1890-03

**Authors:** 


### Editorial.

### THE NATIONAL ASSOCIATION OF DENTAL FACULTIES.

THE question has been raised as to the propriety of changing the time and place of meeting of the Association. It is argued that those members desiring to go abroad the coming summer cannot defer their departure later than the 15th of July, and that, even if they could, the meeting of the American Dental Association, if held at Excelsior Springs, is out of their way. Unless a change is made in the time and place of meeting of the American Dental Association, then it seems that a movement to change the time and place of meeting of the Association of Faculties in order to accommodate those members who desire to attend and also go abroad might well be taken. It is essential that a full representation of the colleges belonging to the association shall be had this year on account of the important measures which will undoubtedly come up to be acted upon, and if the meeting is held in the extreme West, and at the time of the meeting of the American Association, we fear that the attendance will be small, a result very much to be deprecated. The Association did not complete its labors when it voted to establish a three-years' course of instruction. It only entered upon its real work, and it must now see to it that this step in advance is carried out. There are a number of questions yet to be settled in order to set the machinery in motion, and get it to running smoothly.

It is barely possible that some changes may yet have to be made before the measure is accepted by all the colleges connected with the Association. The question has been before the members for a year, and time has been had fully to consider the movement in all its bearings, and there will undoubtedly be some honest objections made to its adoption as it stands which will have to be met and considered, and which will possibly modify the standing resolutions. It is a movement of great importance, and wants to be entered into with caution. We do not desire to be understood as indicating that there will be any backward steps in the matter, but there may need to be certain modifications so as not to work a hardship upon any institution connected with the Association. Great care must be exercised in drawing the lines so as not to force any institution out of the Association. Unanimity of action is absolutely essential for the full success of the measure. The question of the equalization of the fees will come up at this meeting, and is going to require careful consideration not to work an injury. This does not affect the weaker institutions so much as the stronger ones, and they may be the ones to object, especially the State institutions, which, by reason of being connected with State universities and receiving State aid, are not able to control the question of fees. Then, again, the question of *time service* must needs be considered.

Those institutions which have already adopted a lengthened course, if they are compelled to establish a three-years' course, will labor at a considerable disadvantage. For instance, a college having a two-years' course of nine months each is already giving more actual time to their students than an institution which may adopt the prescribed course of three years of five months each. It seems that it would be more equitable for the Association to establish the time actually to be spent in attendance upon lectures, and allow the different institutions to elect how they will apportion the time. Let the minimum time be set at eighteen months actual attendance, and let that time be divided into three years of six months each, or two years of nine months each. This question will undoubtedly be brought before the Association, and presents features that are to be commended. If a nine-months' course should be adopted, the college year could be divided into three terms, of three months each, and students could matriculate at the beginning of any one of these terms, and receive credit for the actual time spent in attendance. A graded course could be established, and an examination made upon the work done, and, if successful, a certificate given which could be presented at any other institution for just what it called for, and credit be given therefor; and when a student could show certificates for eighteen months' attendance, he could come up for final examination, and receive his diploma at the next regular commencement. Such a plan would work a special benefit for those institutions that are short of clinical material, by extending the course over a greater length of time, and thus distributing the year's work more evenly. When the course is limited to five or six months, it draws heavily upon the material at command to supply the needed demand. It would operate also to lessen the number of demonstrators employed, as the class could be divided into smaller sections. Better demonstrators could thus be engaged by employing a less number and extending the time of service to the entire year. Such is, however, only a few of the questions that have been presented to us. The different institutions have their own peculiar difficulties to contend with, and these are more or less aggravated by the proposed compulsory change in the established course, and there will, no doubt, be several knotty questions brought before the Association for its deliberation which will require time and a full attendance wisely to consider and pass upon.

The meeting of the Association of Faculties seriously interfered with the success of the American Dental Association last year by taking from the latter many of its active members, and as there is not the same need of itinerancy upon the part of the Association of Faculties as there is on that of the American Dental Association, would it not be well to call a meeting of the Association of Faculties the first week of July, at some more central point than Excelsior Springs, say at Niagara Falls? This would accommodate those members who desire to go abroad, and insure a full attendance, and not in the least interfere with the meeting of the American Dental Association.

